# Immunogenicity of a West Nile Virus DIII-Cholera Toxin A_2_/B Chimera after Intranasal Delivery

**DOI:** 10.3390/toxins6041397

**Published:** 2014-04-22

**Authors:** Juliette K. Tinker, Jie Yan, Reece J. Knippel, Panos Panayiotou, Kenneth A. Cornell

**Affiliations:** 1 Department of Biological Sciences, Boise State University, Boise, ID 83725, USA; 2 Department of Chemistry and Biochemistry, Boise State University, Boise, ID 83725, USA; E-Mails: jennyyan@boisestate.edu (J.Y.); reeceknippel@u.boisestate.edu (R.J.K.); panospanayiotou@u.boisestate.edu (P.P.); kencornell@boisestate.edu (K.A.C.)

**Keywords:** West Nile virus, cholera toxin, vaccine, mucosal adjuvant

## Abstract

West Nile virus (WNV) causes potentially fatal neuroinvasive disease and persists at endemic levels in many parts of the world. Despite advances in our understanding of WNV pathogenesis, there remains a significant need for a human vaccine. The domain III (DIII) region of the WNV envelope protein contains epitopes that are the target of neutralizing antibodies. We have constructed a chimeric fusion of the non-toxic cholera toxin (CT) CTA_2_/B domains to DIII for investigation as a novel mucosally-delivered WNV vaccine. Purification and assembly of the chimera, as well as receptor-binding and antigen delivery, were verified by western blot, GM1 ELISA and confocal microscopy. Groups of BALB/c mice were immunized intranasally with DIII-CTA_2_/B, DIII, DIII mixed with CTA_2_/B, or CTA_2_/B control, and boosted at 10 days. Analysis of serum IgG after 14 and 45 days revealed that mucosal immunization with DIII-CTA_2_/B induced significant DIII-specific humoral immunity and drove isotype switching to IgG2a. The DIII-CTA_2_/B chimera also induced antigen-specific IgM and IgA responses. Bactericidal assays indicate that the DIII-CTA_2_/B immunized mice produced DIII-specific antibodies that can trigger complement-mediated killing. A dose escalation resulted in increased DIII-specific serum IgG titers on day 45. DIII antigen alone, in the absence of adjuvant, also induced significant systemic responses after intranasal delivery. Our results indicate that the DIII-CTA_2_/B chimera is immunogenic after intranasal delivery and merits further investigation as a novel WNV vaccine candidate.

## 1. Introduction

West Nile virus (WNV) is a positive, single-stranded RNA virus belonging to the arthropod-borne genus *Flavivirus*. Originally isolated in the West Nile district of Uganda in 1937, WNV has spread to other parts of Africa, Asia, Europe and the Middle East, and was first reported in New York in 1999 [1,2]. Virological and ecological factors, including the virulence of isolates and the availability of competent vectors, has permitted WNV to spread rapidly across North America; causing almost 30,000 human cases in the United States between 1999 and 2011. In 2012, there was a dramatic upsurge in WNV transmission in the U.S. resulting in the highest mortality rate recorded in this country, with 2873 cases of neuroinvasive disease and 286 deaths [3,4]. This resurgence is compelling evidence that the virus will remain endemic in the U.S. and continue to trigger sporadic epidemics. 

The majority of humans infected with WNV are asymptomatic or exhibit self-limiting symptoms, but approximately 1% of patients develop neuroinvasive disease that can lead to permanent disability or death [5,6]. Neurological disease is most prevalent and severe in patients over 50 years old that have certain medical conditions, suggesting that host immune status plays an important role in disease outcome [7,8,9]. WNV initially infects dendritic cells (DCs) in the skin at the site of injection and spreads through the bloodstream to other organs, and potentially the CNS [10,11,12]. Protection or control of infection requires both innate and adaptive responses [13,14,15]. Type I interferons (IFN-α/β) are required to control viral replication and dissemination early in infection [16]. However, humoral immunity is essential for viral clearance, and neutralizing IgG and IgM antibodies against WNV surface envelope (E) and premembrane (prM) proteins constitute a majority of the protective response [10,14,16,17,18]. Antibodies directed to WNV E protein block receptor-binding and viral membrane fusion required to release the nucleocapsid into the cytoplasm [19,20]. E protein is subdivided into three domains with the surface-exposed domain III (DIII) producing the most potent neutralizing antibodies [21,22,23].

Vaccine development for WNV remains a top priority as there is no licensed human vaccine, and antiviral therapy has shown limited efficacy [24]. A variety of vaccine paradigms have been explored [25,26]. The human vaccine candidates that have advanced the furthest in clinical trials consist of WNV prM and E proteins expressed in a live attenuated yellow fever or dengue virus background (ChimeriVax-WN02; WN-DEN4). These vaccines have been found to be safe and effective in healthy human volunteers [27,28,29]. Additional live vaccines based on replication-deficient WNV strains, as well as WNV antigens engineered into influenza, adenovirus, and measles delivery systems are in pre-clinical development [30,31,32,33]. Live vaccines are advantageous as they are less expensive to produce and induce long term B and T-cell responses. However, these vaccines are also less safe for the elderly and immunocompromised populations who are most at risk for severe WNV infection. A number of potential subunit vaccines based on recombinant E protein have shown promise in animal models after parenteral delivery [34,35,36,37,38,39]. Mucosal or needle-free administration of these candidate antigens would promote compliance, improve safety and increase access to rural areas of low and middle-income countries. 

The bacterial enterotoxins, including *Vibrio cholerae* cholera toxin (CT) and *Escherichia coli* heat-labile toxin (LTI) have long been recognized as potent adjuvants that can bind to and target immune effector cells at mucosal and dermal sites [40,41,42,43]. CT can act as both a stimulatory and delivery adjuvant, and immunomodulation has been attributed to the ability of CT to activate antigen presenting cells, promote B-cell isotype switching, and upregulate co-stimulatory molecules and MHC class II [44,45,46]. These responses result from the interaction of the pentameric B subunit (CTB) with ganglioside GM1 on effector cells, such as dendritic cells, resulting in antigen uptake and cellular activation [46]. Although toxigenic CT, that comprises CTB and the active A subunit (CTA), is a more potent adjuvant, studies have reported that non-toxic CTB alone can act as an antigen carrier and is immunostimulatory [47,48,49]. Attachment or association of the antigen to CTB will enhance this activity [50]. Holotoxin-like CTA_2_/B chimeras that retain the ganglioside binding activity of CTB and the endoplasmic reticulum-targeting motif within the CTA_2_ domain, but replace the toxic CTA_1_ domain with an antigen of interest, have been developed as mucosal vaccines [51,52]. Evidence suggests that mucosally delivered CTA_2_/B chimeras can activate antigen-specific systemic humoral and cellular immunity, promote protective responses and block the induction of oral tolerance [45,53,54,55,56].

Here we report the construction of a DIII-CTA_2_/B chimeric fusion and the murine immune response to this construct after intranasal delivery. Our results indicate that this novel WNV vaccine can induce DIII-specific systemic immunity after mucosal delivery, and that the CTA_2_/B chimeric configuration is optimal over a mixture of antigen and adjuvant. We also observed that intranasal delivery of WNV DIII antigen alone, in the absence of exogenous adjuvant, can induce significant antigen-specific humoral responses. Both candidates merit further investigation as novel WNV vaccines that will advance the use of alternative routes of delivery. 

## 2. Results

### 2.1. Expression and Characterization of the DIII-CTA_2_/B Chimera

**Figure 1:**
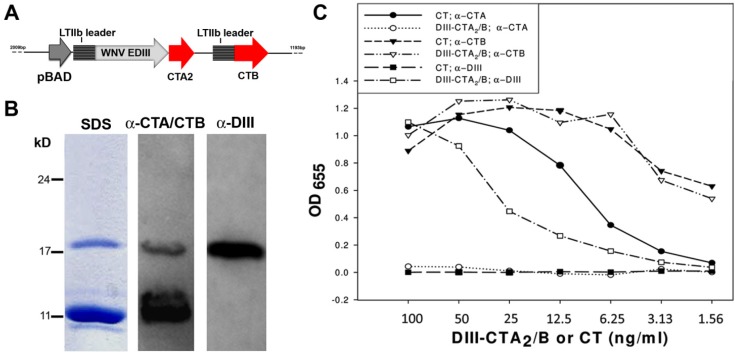
Purification and characterization of the DIII-CTA_2_/B chimera. (**A**) Operon organization of pJY001 for DIII-CTA_2_/B expression; (**B**) SDS-PAGE and western blots (using α-CTA/CTB and α-DIII antibodies) of the DIII-CTA_2_/B chimera purified from a d-galactose affinity column (DIII-CTA_2_ ≈ 18 kD and monomeric CTB ≈ 11.5 kD); (**C**) GM1 ELISA using α-CTA (circles), α-CTB (triangles) and α-DIII (squares) to compare receptor binding activity of DIII-CTA_2_/B (open) with native CT (solid). Mean of duplicate samples (with <10% variation) is shown and results are representative of two independent assays.

The DIII-CTA_2_/B chimera was expressed in *E. coli* from plasmid pJY001 (Figure 1A). This plasmid, constructed from the parental vector pARLDR19, utilizes *E. coli* LTIIB leader sequences to direct expression of the DIII-CTA_2_ fusion protein and monomeric CTB to the periplasm. Subunits fold into holotoxin-like molecules in the *E.coli* periplasm and are purified on d-galactose agarose [57,58]. The CTB subunit will bind the affinity column and co-purification of the CTA_2_ fusion is indicative of holotoxin formation. The resulting yield of DIII-CTA_2_/B chimeric holotoxin was 2–3 mg per 1 liter of starting culture. Holotoxin formation was confirmed by SDS-PAGE and western blot analysis with anti-CTA/CTB and anti-DIII antibodies (Figure 1B) which revealed co-purification of the DIII-CTA_2_ fusion protein (≈18.0 kD) with CTB (≈11.5 kD). To assess receptor-binding activity of the DIII-CTA_2_/B chimera, we performed a ganglioside GM1 ELISA using anti-CTA, anti-CTB and anti-DIII antibodies (Figure 1C). Native CT and DIII-CTA_2_/B were detected at similar levels using anti-CTB in this assay (open/filled triangles). As expected, the DIII antibody was specific for the DIII-CTA_2_/B chimera (open squares) and did not react with native CT (filled squares). The lower anti-CTA response to DIII-CTA_2_/B (open circles) relative to native CT (closed circles) was not unexpected since the chimera contains a small fragment (<10%) of the full length native CTA. Results indicate that the purified DIII-CTA_2_/B chimera assembled a functional CTB pentamer that has ganglioside receptor recognition comparable to native CT. 

**Figure 2:**
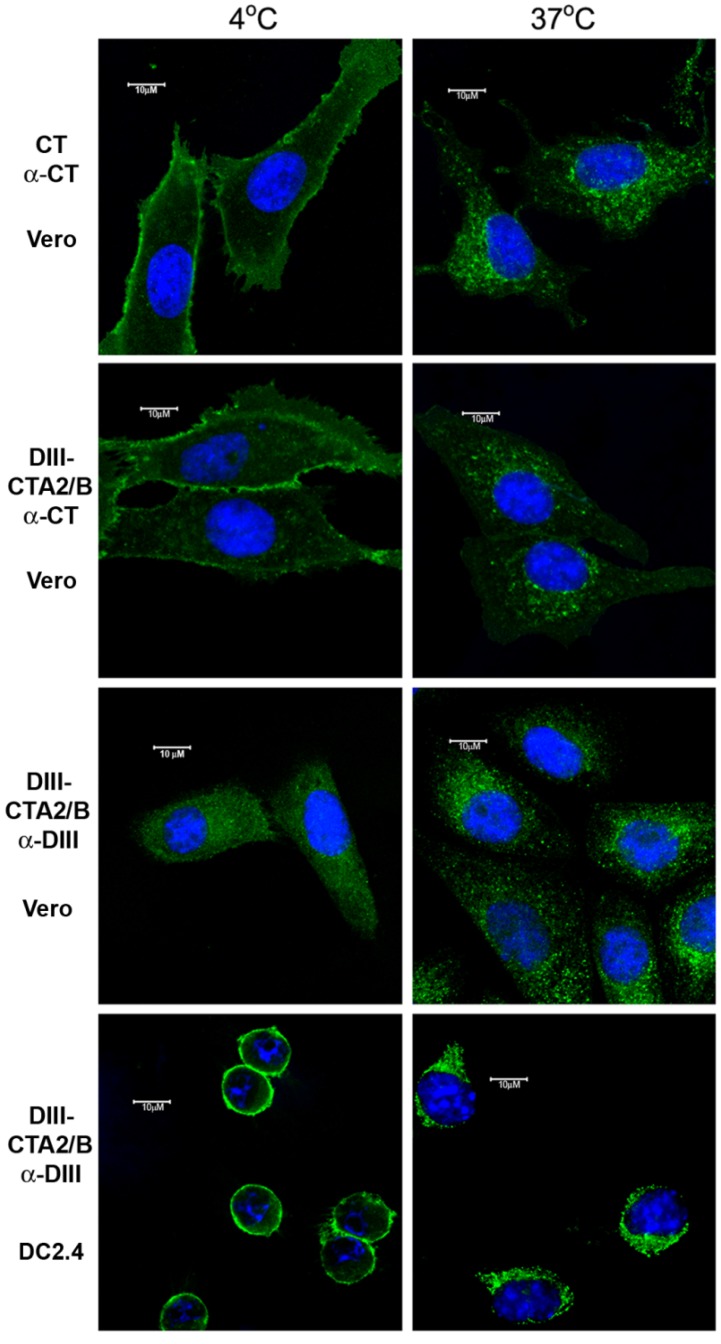
CT and DIII-CTA_2_/B chimera binding and antigen transport. Confocal microscopy of binding and transport into epithelial (Vero) and dendritic (DC2.4) cells. Cells were incubated for 45 min at 4 °C to inhibit, or 37 °C to promote, endocytosis. A FITC-conjugatedsecondary antibody recognized α-DIII or α-CT and cell nuclei were visualized using DAPI staining (100× magnification). Results are representative of at least two independent experiments.

To further characterize both the receptor binding and antigen-delivery capacity of the DIII-CTA_2_/B chimera, *in vitro* binding and trafficking of this molecule on Vero epithelial cells and mouse dendritic DC2.4 cells was investigated using confocal microscopy (Figure 2). Vero cells are well characterized for CT binding and uptake [57,59]. Incubation of native CT or DIII-CTA_2_/B with Vero cells at 4 °C showed CT and the chimera bound to the cell surface. At 37 °C, the DIII-CTA_2_/B chimera was internalized to a perinuclear compartment, similar to trafficking of CT on these cells. Using anti-DIII staining, a similar binding and internalization pattern was seen for the chimera at 4 °C and 37 °C on both Vero cells and DC2.4 cells indicating the effective uptake of DIII antigen from DIII-CTA_2_/B into these different cell types.

### 2.2. Immunogenicity after Intranasal Delivery

**Figure 3:**
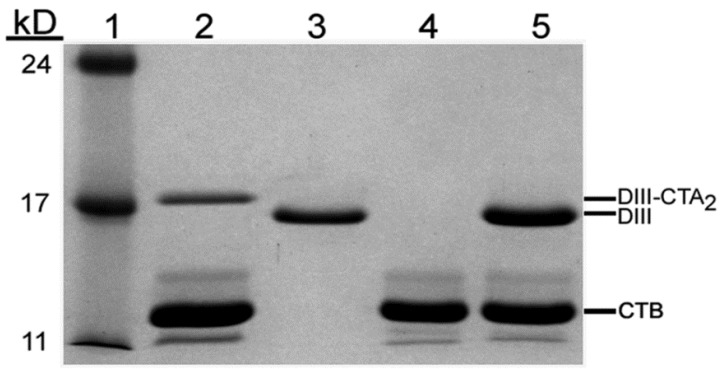
SDS-PAGE of vaccine antigens. (1) molecular weight standards; (2) d-galactose affinity purified DIII-CTA_2_/B; (3) nickel-affinity purified DIII; (4) d-galactose affinity purified CTA_2_/B; and (5) mixture of DIII + CTA_2_/B. 10 μg of protein loaded per lane.

A primary immunogenicity study was performed in which groups of 6 mice were inoculated with 50 μg of DIII-CTA_2_/B or a molar equivalent of purified DIII alone (15 μg), mixed DIII (15 μg) + CTA_2_/B (35 μg), or control CTA_2_/B (35 μg) on day 0 and boosted with the same dose on day 10. SDS-PAGE of the antigen preparations used in the immunization confirmed expected composition and purity of the vaccines (Figure 3). 

**Figure 4:**
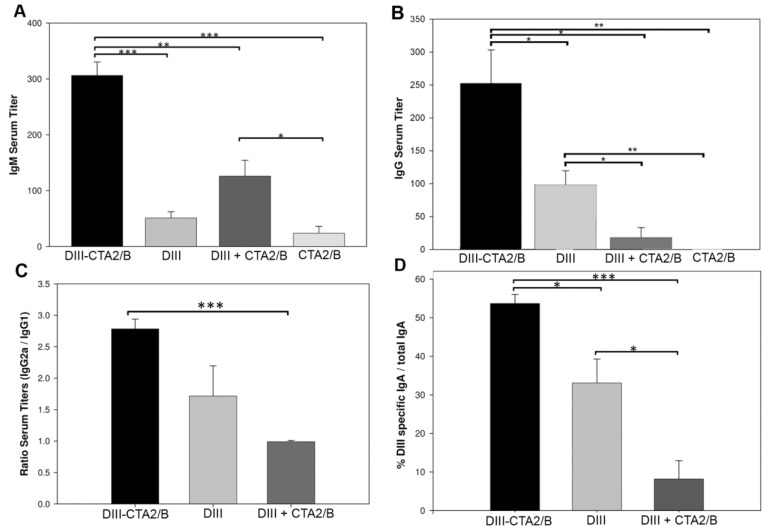
Immune responses to DIII-CTA_2_/B intranasal vaccination. (**A**) DIII-specific IgM ELISA endpoint titers from day 14 sera pooled by immunization group; (**B**) DIII-specific IgG ELISA endpoint titers from day 14 sera pooled by immunization group; (**C**) Ratio of DIII-specific IgG2a to IgG1 ELISA endpoint titers from day 45 sera pooled by immunization group; (**D**) Percent DIII-specific mucosal IgA out of total IgA in day 45 nasal wash as determined by IgA ELISA (1:40 dilution) of wash pooled by immunization group (*n* = 6). Results are presented as the mean (±SEM) of three independent assays. Significance: *p* < 0.05(*), *p* < 0.01(**) or *p* < 0.001(***), between immunization groups for all assays is shown.

Serum collected on days 14 and 45 was pooled by treatment group and tested for the presence of anti-DIII antibodies by ELISA. DIII-specific serum IgM responses were analyzed at day 14, and results revealed a significant induction in the DIII-CTA_2_/B immunized group over control groups (Figure 4A). The mixed DIII + CTA_2_/B group also induced significant DIII-specific IgM responses relative to the CTA_2_/B control. On day 14, DIII-specific serum IgG endpoint titers from mice immunized with the DIII-CTA_2_/B chimera were significantly higher as compared with all other groups, and IgG titers from DIII immunized mice were also significantly higher than mixed DIII + CTA_2_/B and CTA_2_/B control immunized mice (Figure 4B). Background IgM and IgG level responses (similar to day 0) were assayed from the CTA_2_/B only control immunized group on day 14. 

DIII- specific IgG subclasses from day 45 serum were further assessed after subtraction of control group, and the ratio of IgG2a to IgG1 determined (Figure 4C). Animals in the DIII-CTA_2_/B chimera immunized group showed an IgG2a/IgG1 ratio that indicated isotype switching toward the IgG2a subclass and suggested promotion of a Th1-type cellular response. The IgG2a/IgG1 ratio in sera from mice immunized with DIII alone and DIII + CTA_2_/B mixed were not significantly different and indicated a mixed Th1/Th2 response. 

To assess the mucosal response which is characteristic of intranasal CT as an adjuvant, nasal washes were collected on day 45 pooled by treatment group, and tested for recognition of DIII by IgA ELISA (Figure 4D). These results also reveal that the DIII-specific IgA response was significantly higher in nasal washes from mice immunized with DIII-CTA_2_/B compared to the DIII alone and mixed DIII + CTA_2_/B treatment groups. DIII alone also stimulated DIII specific mucosal IgA responses that were significantly higher than the mixed DIII + CTA_2_/B group. 

**Figure 5:**
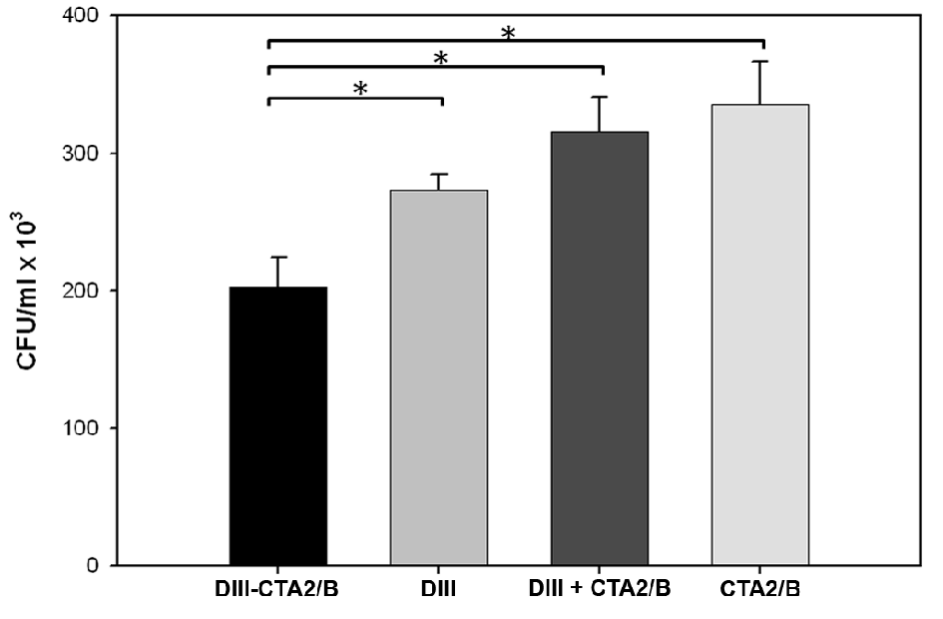
Complement-mediated bactericidal assay of serum from immunized mice. Pooled, heat-inactivated, day 45 serum (1:50 dilution) from immunized mice was mixed with *E. coli* expressing DIII antigen in the presence of rabbit complement. The results shown are the mean (±SEM) of two independent assays performed in duplicate. Significance: *p* < 0.05(*) between immunization groups is shown.

To measure the *in vitro* functional activity of DIII-specific antibodies from immunized mice, serum bactericidal assays using a strain of *E. coli* that expresses DIII in the periplasm and supernatant were performed (Figure 5). DIII expressing bacteria were incubated with pooled, heat-inactivated, day 45 sera from immunized mice and rabbit complement to measure antibody-stimulated complement killing. The assay demonstrated that sera from DIII-CTA_2_/B immunized mice can activate complement and significantly reduce CFU of DIII-expressing bacteria compared to control groups. 

**Figure 6:**
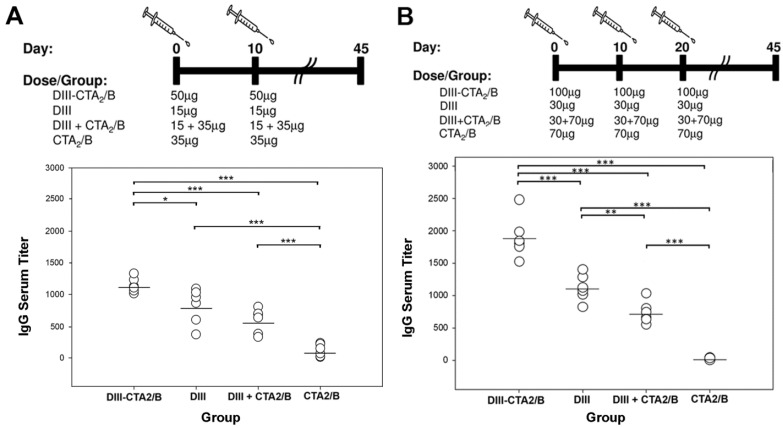
Day 45 humoral responses from two vaccination schemes. (**A**) Vaccination schedule and dosage for the primary vaccination protocol (*n* = 6) and DIII-specific endpoint IgG titers from day 45; (**B**) Vaccination schedule and dosage for the secondary vaccination protocol (*n* = 6) and DIII-specific endpoint IgG titers from day 45. Results are presented as the mean titer of individual mice from three independent assays. Significance: *p* < 0.05(*), *p* < 0.01(**) or *p* < 0.001(***), between immunization groups is shown

A second study was performed in mice to assess the safety and immunogenicity of increased vaccine dosage. 100 μg of the DIII-CTA_2_/B chimera and molar equivalents of DIII, DIII + CTA_2_/B and CTA_2_/B were inoculated intranasally in mice on days 0, 10 and 20. Sera from individual mice were assayed for DIII-specific endpoint titers from day 45 and compared to the primary study (Figure 6). Consistent with day 14, individual endpoint titers from day 45 of the primary study reveal responses from the DIII-CTA_2_/B vaccinated mice that were significantly higher than DIII responses in all other groups and the immunogenicity of DIII alone was significantly higher than mixed and control groups (Figure 6A). Results from the secondary study reveal a consistent pattern between groups with a significant induction of DIII-specific responses in the DIII-CTA_2_/B vaccinated group over all other groups. (Figure 6B). With the addition of a second boost, overall titers from the DIII-CTA_2_/B, DIII alone and DIII + CTA_2_/B groups were higher in the secondary study with no adverse effects. 

In summary, intranasal immunization with the DIII-CTA_2_/B chimera stimulated antigen-specific serum IgG and IgM responses, and secretory IgA, that were significant over responses in animals immunized with antigen alone, mixed antigen and adjuvant (DIII + CTA_2_/B), or adjuvant alone (CTA_2_/B). Immune responses were indicative of Th1-type and produced antibodies that were functionally able to activate complement *in vitro*. Unexpectedly, intranasal immunization with purified DIII antigen, in the absence of additional adjuvant, was also effective in stimulating significant systemic IgG responses that were improved with increased dosage. 

## 3. Experimental Section

### 3.1. Ethics Statement

All animal protocols were approved by the Institutional Animal Care and Use Committee at the Veterans Affairs Medical Center in Boise, Idaho or by the Boise State University IACUC. The Boise VA Medical Center is accredited by the American Association for Accreditation of Laboratory Animal Care (AAALAC), and both the Boise VA and the Boise State University facilities are registered with the USDA and approved by the Office of Laboratory Welfare (OLAW).

### 3.2. Bacterial Strains

*Escherichia coli* TE1, a ∆*endA* derivative of TX1, Origami (Novagen, Madison, WI, USA) and BL21(DE3)pLysS (Invitrogen, Carlsbad, CA, USA) were used for protein expression or bactericidal assays. All strains were grown in Luria-Bertani (LB) or Terrific Broth (TB) at 37 °C with chloramphenicol (35 μg/mL), ampicillin (100 μg/mL), and/or kanamycin (50 μg/mL) as selection agents.

### 3.3. Plasmid Construction

WNV NY99 strain mRNA isolated from infected mosquitoes was the kind gift of Dr. Chris Ball (Idaho Bureau of Labs). Viral cDNA was synthesized using an Invitrogen SuperScript cDNA Synthesis kit. To construct the DIII-CTA_2_/B chimera, the sequence encoding DIII of WNV E protein (encoding amino acids 291-406) was PCR amplified from cDNA and cloned into the CT chimera expression vector pARLDR19, via *SphI* and *ClaI* restriction sites to generate pJY001 (primers: GACTGGGCATGCATTGCAGTTGAAGGG and GACTGCATCGATGTTGTAAAGGCTTTG). The pARLDR19 parental vector has been described and contains *ctx*A_2_ and *ctx*B with *E. coli* heat-liable toxin type IIb (LTIIb) leader sequence [57]. For expression of DIII protein alone (amino acids 295-410), the gene sequence was amplified (primers: CACCCTCGAGAAAAGAGGAACAACCTATGGCGTCTGT

TCAAAG and AAAGCGGCCGCTTACGCTCCTTTGAGGGTGGTTGTAAAGGC), and cloned into the pET200-TOPO vector (Invitrogen) to make the plasmid pET-DIII. Plasmid pJY012A was constructed by cloning the DIII gene sequence (primers: AAAAGTACTTTGCAGTTGAAGGGAACAACCTATG and ATAAGAATGCGGCCGCTGTAAAGGCTTTGCCAATGC) into the pET40b(+) vector (Novagen, Madison, WI, USA) via *ScaI* and *NotI*, to express DIII (amino acids 291-406) in *E.coli* BL21(DE3) pLysS for bactericidal assays. All plasmids were sequenced to confirm fidelity (SeqWright, Houston, TX, USA). 

### 3.4. Expression and Purification of Proteins

To purify the DIII-CTA_2_/B chimera, *E. coli* Origami with pJY001 was grown in TB to an OD600 = 0.9, and induced with 0.2% L-arabinose for 15 h. Proteins were purified from the periplasmic extract and supernatant using immobilized d-galactose as described previously [58]. To purify CTA_2_/B, TE1 cells with pARLDR19 were grown, induced and the periplasmic extract was purified as above for DIII-CTA_2_/B. For vaccinations and ELISAs, recombinant DIII was expressed using isopropylthiogalactoside (IPTG) induction of pET-DIII transformed BL21(DE3) pLysS cells. Briefly, a 25 mL overnight stock culture (37 °C, 225 rpm, LB supplemented with 1% glucose + 50 μg/mL kanamycin[LBGkan^5^°]) was diluted into 1 L fresh LBGkan^5^° broth and incubated with shaking at 37 °C until the culture reached midlog phase (OD_600_ ≈ 0.4–0.7). Expression was induced with IPTG (0.5 mM final) overnight at room temperature. Cells were harvested by centrifugation and washed with Cobalt column buffer A (50 mM Na phosphate, pH 7.4, 300 mM NaCl, 10 mM imidazole). Cell pellets were resuspended in 10 mL buffer A and lysed by sonication. Insoluble debris was removed by centrifugation and the supernatant was applied to a 3 mL Cobalt-resin column (Thermo Scientific, Rockford, IL, USA) equilibrated in buffer A. Recombinant hexahistidine tagged proteins were eluted with 3 column volumes of buffer B (buffer A + 500 mM imidazole), and buffer exchanged into PBS (pH 7.2) using PD-10 columns (GE Healthcare, Pittsburgh, PA, USA). Recombinant proteins were treated with Detoxi-Gel resin (Thermo Scientific Pierce, Waltham, MA, USA) to remove contaminating endotoxin. Prior to use in vaccination schemes, all proteins were assayed using the *Limulus* Amoebocyte Lysate assay (Associates of Cape Cod Pyrotell) and shown to be below the level of detection for endotoxin contamination (sensitivity 0.06 EU/mL). The identity of all proteins was further confirmed by liquid chromatography/mass spectrometry (LC/MS) at the Boise State University mass spectrometry facility. Final protein concentrations were determined by UV spectroscopy (DIII A_280_ 0.1% = 0.709) or BCA assay (Thermo Scientific). Protein aliquots in 5% glycerol were stored at −80 °C until use in vaccine or ELISA studies. 

### 3.5. Protein Analysis and Western Blot

Purified proteins were boiled in protein sample buffer with β-mercaptoethanol and analyzed by 12% SDS-polyacrylamide gel electrophoresis (SDS-PAGE). Proteins in SDS-PAGE gels were stained with Coomassie Blue or transferred to nitrocellulose membranes. For western blot analysis, membranes were incubated with blocking buffer (0.05% Tween-20 + 5% skim milk in PBS) overnight at 4 °C and probed with rabbit anti-CTA polyclonal antibody (1:2000, kindly supplied by R.K. Holmes), rabbit anti-CTB polyclonal antibody (1:5000, Abcam, Cambridge, MA, USA) or mouse anti-WNV DIII monoclonal antibody 7H2 (1:2000, BioReliance, Rockville, MD, USA) diluted in blocking buffer. HRP-conjugated mouse anti-rabbit IgG (1:5000, Promega, Madison, WI) or HRP-conjugated goat anti-mouse IgG (1:3000, Thermo Scientific) was used as the secondary antibody. Blots were developed using Immobilon Western HRP substrate (Millipore, Billerica, MA, USA). 

### 3.6. Ganglioside GM1 ELISA Assay

The GM1 ganglioside binding assay was performed as described previously [58]. Briefly, 96-well plates were coated with 100 µL of 1.5 µM GM1 (Alexis Biochemicals, San Diego, CA, USA) overnight at room temperature. Plates were treated with ELISA blocking buffer (PBS + 10% bovine serum albumin) at 4 °C overnight and incubated with two-fold serial dilutions of native CT or DIII-CTA_2_/B in 100 µL blocking buffer for 1 hour at room temperature. Wells were incubated with anti-CTA antibody (1:2000), anti-CTB antibody (1:5000) or mouse anti-WNV DIII monoclonal antibody 7H2 (1:2000) diluted in blocking buffer for 1 hour, washed three times with PBS + 0.05% tween-20 , and incubated with HRP-conjugated mouse anti-rabbit IgG (1:5000) or HRP-conjugated goat anti-mouse IgG (1:3000) for 1 hour at room temperature. The assay was developed with TMB-one reagent (Promega) and the absorbance was measured at 655 nm. 

### 3.7. Cell Culture and Confocal Microscopy

Green monkey kidney cells (Vero, ATCC) were grown in Dulbecco’s modified Eagle medium (DMEM high glucose, Thermo Scientific) with L-glutamine supplemented with 10% bovine growth serum (BGS) and pen/strep. C57Bl/6 murine dendritic cells (DC2.4, kindly supplied by Kenneth L. Rock, DFCI, Boston, MA, USA) were maintained in RPMI 1640 medium with L-glutamine supplemented with 10% BGS, 10 mM HEPES, 55 µM 2-mercaptoethanol, 1× non-essential amino acid and pen/strep at 37 °C and 5% CO_2_. Internalization assays were performed as described previously [58]. Vero or DC2.4 cells were grown for 24 to 48 h to subconfluence on uncoated coverslips. Coverslips were washed with PBS and incubated at 4 °C for 5 min to slow endocytosis, followed by incubation with 2.5 μg DIII-CTA_2_/B or native CT in 50 μL PBS at 4 °C for 15 min before shifting some cells to 37 °C for 45 min. Cells were fixed with 3.7% formaldehyde, permeabilized with 0.1% triton X-100 in PBS and blocked with 5% bovine growth serum (BGS) in PBS. Rabbit anti-CT polyclonal antibody (1:15000, Sigma, St. Louis, MO, USA) or mouse anti-WNV DIII monoclonal antibody clone 7H2 (1:250) was used as primary antibody, FITC-conjugated anti-rabbit IgG (1:5000, Sigma) or FITC-conjugated anti-mouse IgG-IgM-IgA (1:1000 Abcam) was used as secondary antibody, respectively. Cells were washed several times with PBS + 0.05% tween-20 between steps. Coverslips were mounted with hardset medium with DAPI (Vector Laboratories, Burlingame, CA, USA). Images were acquired using a Zeiss LSM 510 META laser scanning confocal microscope equiped with a 100× Alpha Plan-Fluor 1.45oil DIC objective. 

### 3.8. Mouse Immunizations and Sample Collection

Female 7 to 9 week old BALB/c mice were obtained from Taconic (Oxnard, CA, USA). For the primary immunogenicity study, groups of 6 mice were lightly anesthetized and immunized intranasally with 20 μL PBS (10 μL per nare) containing equimolar concentrations of DIII-CTA_2_/B (50 µg), DIII alone (15 µg), DIII + CTA_2_/B (15 μg + 35 μg) or CTA_2_/B (35 µg) on day 0 and day 10. Antigen doses were based on previous studies [53,56]. Blood samples were collected from the lateral tail vein on day 0, day 14 and by cardiac puncture on day 45. Blood was incubated for 1 hour at room temperate and centrifuged at 4000 ×g for 10 min at 4 °C to separate serum. Serum was diluted 10 fold in buffer containing 5% glycerol and Halt Protease Inhibitor (Thermo Scientific) in PBS. Mucosal fluids were obtained from nares by tying the inferior trachea and washing the superior trachea with 1 mL lavage solution (1× Halt Protease Inhibitor, 5 mM EDTA and 0.02% NaN_3_ in PBS). For the secondary immunogenicity (dose escalation) study, groups of 6 mice were anesthetized and immunized intranasally with 20 μL PBS containing equimolar concentrations of DIII-CTA_2_/B (100 µg), DIII alone (30 µg), DIII + CTA_2_/B (30 + 70 µg) or CTA_2_/B (70 µg) on day 0, day 10 and day 20. Blood was collected on day 0 from the lateral tail vein and on day 45 by cardiac puncture, and processed as above for ELISA analysis. 

### 3.9. IgG, IgM and IgA ELISAs

ELISAs were performed as described previously [56]. Briefly, 96-well plates (Nunc 439454, Rochester, NY, USA) were coated with 10 µg/mL of purified DIII, blocked with 5% BGS in PBS overnight at 4 °C, and washed in PBS-T (1XPBS, 0.05% Tween-20). Plates were then incubated with pooled or individual mouse sera or pooled mucosal fluids diluted in blocking buffer overnight at 4 °C. Plates were washed with PBS-T and incubated for 1 hour at 37 °C with HRP-conjugated goat anti-mouse IgG (1:10000, Thermo Scientific Pierce), IgG1 (1:2000, MP Biomedicals), IgG2a (1:2000, MP Biomedicals), IgM (1:5000, Thermo Scientific Pierce) or rabbit anti-mouse IgA (1:1500 Southern Biotech, Birmingham, AL, USA) secondary antibody in blocking buffer. Assays were developed with TMB One (Promega) and *A*370 nm absorbance values collected using a Synergy HT microplate spectrophotomer (BioTek). To quantify total IgA for mucosal IgA ELISAs, 0.005 µg of rabbit anti-mouse IgA (Thermo Scientific) was used to coat plates. Percent DIII-specific IgA from total IgA was determined after subtraction of the average CTA_2_/B group value to account for variations in mucosal sampling. For IgG and IgM assays, endpoint titers were determined after the subtraction of background (protocol minus serum) for all groups. For IgG2a and IgG1 determination, endpoint titers were determined after subtraction of the average CTA_2_/B group value as background and ratios are based on titers. Endpoint titers for all assays were defined as the reciprocal of the last sample dilution giving an absorbance of 0.1 *A*370 units above background. 

### 3.10. Serum Bactericidal Assays

*E. coli* BL21(DE3) pLysS cells containing plasmid pJY012 were grown in LB broth to an A600 = 0.6–0.9 and induced with 0.1 mM IPTG for 3 hours. Expression of DIII in the supernatant and periplasmic fractions of this strain was confirmed by western blot (data not shown). DIII-expressing *E. coli* (8 × 10^4^ cells/well) were incubated with heat-inactivated pooled mouse serum (1:50 dilution) and activated or inactivated rabbit complement (MP Biomedicals) at 37 °C for 1 hour. Dilutions of the reaction were prepared and plated on LB agar with kanamycin (50 µg/mL) and chloramphenicol (35 µg/mL) and the number of colony-forming units (CFU/mL) determined after overnight incubation at 37 °C. Assays performed using inactivated complement and serum from day 0 showed no significant difference between groups [‎60]. 

### 3.11. Statistical Analysis

IgG, IgM and IgA ELISA’s were performed on pooled or individual serum or pooled nasal wash three independent times. GM1 ELISA and bactericidal assays were performed two independent times in duplicate. Results are expressed as the mean ± the standard error of the mean (SEM) between three independent experiments unless otherwise noted. Comparisons between immunization groups were made using the unpaired student’s t-test. A *p*-value < 0.05 (*), <0.01 (**) or <0.001 (***) was considered a statistically significant difference. Graphing and statistical analyses were performed using SigmaPlot 8.0 (Systat Software).

## 4. Discussion and Conclusions

The goal of the present study was to purify a holotoxin-like WNV DIII-CTA_2_/B chimeric fusion and characterize its immunogenicity after intranasal administration to mice. We have demonstrated that the DIII-CTA_2_/B chimera can be expressed and purified efficiently from *E. coli* and that this molecule retains the ability to bind to mammalian host cells *in vitro* and internalize antigen. Ganglioside GM1 is found ubiquitously on mammalian cells, but immune effector cells, such as dendritic cells, are uniquely targeted and activated by CT and the non-toxic CTB subunit [61,62]. The binding and transport of the DIII antigen from DIII-CTA_2_/B into Vero epithelial and DC2.4 dendritic cells was consistent with the uptake of native CT involving retrograde movement to the perinuclear domain of the Golgi apparatus and endoplasmic reticulum [58,59,63]. We hypothesize that the ability of DIII-CTA_2_/B to bind to GM1 and trigger antigen internalization could lead to the activation of immune effector cells and promote adjuvanticity through antigen presentation on MHC molecules. 

To assess the immune response stimulated by mucosal delivery of the DIII-CTA_2_/B chimera, mice were immunized intranasally, boosted on day 10 and evaluated on days 14 and 45. DIII-specific IgG titers from DIII-CTA_2_/B immunized mice were significantly elevated over groups that included antigen alone, a mixture of antigen plus adjuvant and adjuvant alone as a negative control. These responses are consistent with the ability of previously reported CTA_2_/B chimeras to promote antigen-specific systemic IgG from mucosal sites [52,53,56,64,65]. Antigen-specific IgM levels, which contribute to protection against WNV in early infection, were also stimulated by immunization with the DIII-CTA_2_/B chimera from day 14 serum [14]. Antigen-specific IgG responses were the highest on day 45 of the study. Further analysis of the systemic response indicated that higher levels of IgG2a over IgG1 were stimulated by DIII-CTA_2_/B, indicative of a Th1-type response. This is in contrast to previous reports that CT and CT derivatives promote more of a Th2-type response [46,56,66,67]. However, CTA_2_/B chimeric fusions have been reported to have distinct properties depending largely on the associated antigen, and some have been found to stimulate IgG2a responses, including one developed specifically against respiratory syncytial virus (RSV) [68]. A number of additional reports have shown that CTA_2_/B chimeras can induce significant T-cell responses to fused bacterial and protozoal antigens [53,56,69,70]. This is a desirable response for a WNV vaccine and will continue to be explored. Bactericidal assays were supportive of antigen-specific serum results as the ability of sera to fix complement and lyse bacteria expressing DIII was higher in DIII-CTA_2_/B immunized mice. Previous reports have demonstrated a role for antibody-dependent complement activation in West Nile viral particle neutralization [71]. While our results indicate that antibodies against DIII are functional *in vitro,* extrapolation to viral neutralization or *in vivo* protection cannot be done at this early stage, and these studies will be pursued in the future. 

To build upon the above results, we performed a dose escalation with a higher concentration of vaccine and a second boost on day 20. Results from the secondary study supported our initial findings; showing a significant stimulation of DIII-specific responses on day 45 in the DIII-CTA_2_/B vaccinated mice over other groups, and a higher overall response with increasing vaccine dose. In addition, this study supported evidence that intranasal delivery of DIII alone, in the absence of extrinsic adjuvant, could also induce significant antigen-specific responses. These unexpected results indicate that WNV DIII possess a potential intrinsic immunostimulatory or antigen-delivery activity. Recombinant DIII antigen has previously been found to be immunogenic after intraperitoneal or subcutaneous delivery in the presence of adjuvant [35,37,72,73]. Chu *et al.* (2007) also identified responses to recombinant DIII after intraperitoneal injection in the absence of adjuvant [36]. To our knowledge, the delivery of DIII antigen alone via the intranasal route has not been reported. The DIII region is proposed to play a role in viral entry and infection efficiency through interactions with α_v_β_3_ integrins, in a manner that is independent of the classic arginine-glycine-aspartic acid (RGD) binding motif [74,75,76]. Others have reported that WNV entry into cells is dependent on cholesterol-rich lipid rafts [77]. Since α_v_β_3_ integrin has been reported on a subset of murine dendritic cells and to be involved in adenoviral mediated antigen delivery, it is possible that our findings reflect DIII antigen binding, uptake and processing via this cellular receptor [78,79]. Alternatively, the DIII antigen may be interacting with α_v_β_3_ integrin to direct the protein to cellular entry via cholesterol-rich rafts and dynamin, as has been reported for herpes simplex virus [80,81]. Thus, it is possible that the DIII antigen alone can stimulate protective immune responses at higher concentrations in the absence of traditional adjuvants. 

The mix of purified DIII with CTA_2_/B failed to stimulate a significant IgG response at day 45 and only stimulated a small IgM response at day 14. These results are in contrast to previous studies that have shown that a mixture of antigen and CTA_2_/B is as good as chimera at inducing antigen-specific IgG on day 45 [56]. It is possible that the binding of the individual DIII and CTA_2_/B components in this study to separate cellular receptors (α_v_β_3_ integrin/lipid rafts *vs.* GM1) may have antagonized subsequent antigen delivery. Alternatively, the low humoral responses observed from the mixed DIII + CTA_2_/B group may be explained by an ineffective assembly of antigen and adjuvant in this preparation. CTB has been shown to induce immunological unresponsiveness or tolerance to certain types of mucosally co-administered antigens depending on the antigen and the dosage, as well as the antigen-adjuvant configuration [53,82]. While CTA_2_/B chimeras have been reported to abrogate oral tolerance for up to 6 months, studies have also shown that chemical conjugation or genetic fusion of antigens directly to CTB can induce unresponsiveness [53,83]. An aggregation between DIII and CTA_2_/B may have occurred in our mixed preparation, resulting in a configuration that induced tolerance by day 45 rather than immune stimulation. While we saw significant immune stimulation with the DIII-CTA_2_/B chimera after 45 days, the potential for the induction of long term immunological tolerance by this antigen and delivery route will continue to be assessed. 

CT and the closely related LTI are the gold standard for mucosal adjuvants, with a long history of use in animals. However, the safety of mucosal administration of these toxins in humans has been questioned due to reports that they can redirect antigens to the central nervous system through GM1-dependent binding to olfactory epithelium [84,85,86]. Despite the elimination of the toxic domain in A_2_/B chimeras, safety concerns still remain. A brief licensure of an influenza vaccine and more recent clinical study of non-toxic LTI support a connection to facial nerve paralysis after intranasal delivery [87,88]. Oral, sublingual or transdermal vaccination with CTB however, does not target olfactory neurons, and does not raise the same safety concerns [89]. CTB is a component of the current oral *V. cholerae* vaccine WC-rBS (Dukoral, Crucell) that is licensed in over 60 countries, is well tolerated, and has a good safety record [90]. As proof-of-principle in this report, we have chosen the intranasal route that is well characterized, requires a lower concentration of antigen and has been reported previously for CTA_2_/B chimeras. Safe and effective administration of CT and LTI-based vaccines by oral, sublingual, and transcutaneous routes has proven promising in mice and humans, and may also prove to be effective for DIII-CTA_2_/B [54,91,92,93,94,95,96,97]. 

To our knowledge, the construction and characterization of a mucosally delivered antigen is a unique approach for WNV vaccine development. We report the expression and purification of a DIII-cholera toxin chimeric fusion and the induction of antigen-specific humoral responses in micefollowing intranasal delivery of this construct. In addition, we report the novel finding of DIII antigen immunogenicity in mice after mucosal delivery in the absence of adjuvant. Both DIII and DIII-CTA_2_/B warrant further investigation as novel WNV subunit vaccines. 
